# Regulation of Structure and Anion-Exchange Performance of Layered Double Hydroxide: Function of the Metal Cation Composition of a Brucite-like Layer

**DOI:** 10.3390/ma15227983

**Published:** 2022-11-11

**Authors:** Luwen Tang, Xiangli Xie, Cunjun Li, Yanqi Xu, Wenfeng Zhu, Linjiang Wang

**Affiliations:** 1 College of Materials Science and Engineering, Guilin University of Technology, Guilin 541004, China; 2 College of Mechanical and Control Engineering, Guilin University of Technology, Guilin 541004, China; 3 Guangxi Key Laboratory of New Energy and Building Energy Saving, Guilin University of Technology, Guilin 541004, China; 4 College of Chemistry and Bioengineering, Guilin University of Technology, Guilin 541004, China; 5 Key Laboratory of New Technology for Processing Nonferrous Metals and Materials, Ministry of Education, Guilin University of Technology, Guilin 541004, China; 6 Collaborative Innovation Center for Exploration of Nonferrous Metal Deposits and Efficient Utilization of Resources in Guangxi, Guilin University of Technology, Guilin 541004, China

**Keywords:** layered double hydroxide, metal cation composition, structure regulation, anion-exchange performance, interlayer domain, intercalation driving force

## Abstract

As anion-exchange materials, layered double hydroxides (LDHs) have attracted increasing attention in the fields of selective adsorption and separation, controlled drug release, and environmental remediation. The metal cation composition of the laminate is the essential factor that determines the anion-exchange performance of LDHs. Herein, we review the regulating effects of the metal cation composition on the anion-exchange properties and LDH structure. Specifically, the internal factors affecting the anion-exchange performance of LDHs were analyzed and summarized. These include the intercalation driving force, interlayer domain environment, and LDH morphology, which significantly affect the anion selectivity, anion-exchange capacity, and anion arrangement. By changing the species, valence state, size, and mole ratio of the metal cations, the structural characteristics, charge density, and interlayer spacing of LDHs can be adjusted, which affect the anion-exchange performance of LDHs. The present challenges and future prospects of LDHs are also discussed. To the best of our knowledge, this is the first review to summarize the essential relationship between the metal ion composition and anion-exchange performance of laminates, providing important insights for regulating the anion-exchange performance of LDHs.

## 1. Introduction

Anion-exchange performance is an essential characteristic and the basis for layered double hydroxides (LDHs), critical in the application of LDHs as selective materials for adsorption and separation, drug control and release, and environmental remediation [1,2,3]. The LDH structure is an ordered assembly of positively charged main laminates and interlayer anions. The general formula for the chemical composition of LDHs is [M^II^_1-x_M^III^_x_(OH)_2_]^x+^ [A_x/n_]^n−^·mH_2_O, in which the types of divalent M^II^ and trivalent M^III^ metal cations can be adjusted on a wide scale. Because the positive charge of the main laminate is obtained through the isomorphic substitution of divalent with trivalent metal cations, the quantity and distribution of the positive charge of the main laminate can be adjusted by changing the cation type and molar ratio x. Thus, the anions A^n−^ enter the LDH interlamination mainly by relying on the positive charge. In addition to the common M^II^−M^III^ LDHs, other variations such as M^I^−M^III^ LDHs (Li−Al LDHs) [4], M^II^−M^IV^ LDHs (e.g., M^IV^ = Ti^4+^, Zr^4+^, and Sn^4+^) [5], and M^I^−M^II^ LDHs (Na−Ca LDHs) [6] have been reported.

Owing to their anion-exchange performance, LDHs are considerably attractive for the construction of intelligent and high-performance multifunctional materials [7]. This is because: (1) a wide variety of anions can be intercalated into the LDH interlayers, thereby providing more modification possibilities of the materials; (2) the intercalation of guest anions can cause lattice expansion and increase the anion-exchange capacity; (3) the anion-exchange process and its accompanying property changes are usually reversible; (4) the anion-exchange process is controllable, including the type, quantity, and size of intercalated anions; and (5) the intercalation of guest anions adds new degrees of freedom for the adjustment of LDHs, which can be combined with other modification methods. Therefore, the anion-exchange performance of LDHs must be improved for a better understanding of the mineralogical characteristics of anionic clay minerals and their application in the field of advanced mineral materials.

To date, studies on the regulation of the LDH anion-exchange performance have mainly focused on three aspects: (1) improving the guest anion interlayer driving force; (2) changing the interlayer domain environment; and (3) optimizing the LDH morphology [8]. There are various regulation methods, including changing the external environmental factors (e.g., temperature, pressure, and solution pH), LDH synthesis methods, and lamellar cation composition [9]. However, the regulation effect of the lamellar metal ions on the anion-exchange performance of LDHs has not been systematically recognized. In essence, the composition of the lamellar metal ions is the foundation of the structure and is critical for the performance of an LDH, and the synthesis methods and external environmental conditions are changed based on this foundation and the application purpose. The type, structure, and quantity of anions entering the interlayer of LDHs are determined by the environmental characteristics of the interlayer domain, which in turn are controlled by the structural characteristics of the main laminates of the material. In particular, the charge density characteristics of the laminates are determined by their metal cation composition, which is therefore key to determining the interlayer domain environment and anion-exchange performance.

With these facts in mind, this review mainly focuses on the regulating effects of the metal cation composition on the anion-exchange performance of LDHs. Special attention was paid to the regulation of the metal cation composition on the intercalation driving force of the guest anion, interlayer domain environment, and LDH morphology. In addition, the anion-exchange performance of LDHs in applications, such as the preparation of new materials, pollutant removal, and as drug carriers are overviewed. Finally, the future research direction of LDH materials in anion exchange is explored.

## 2. Regulation Effect of the Anionic Intercalation Driving Force

### 2.1. Effect of the Anion Intercalation Driving Force on the Anion-Exchange Performance

**Figure 1:**
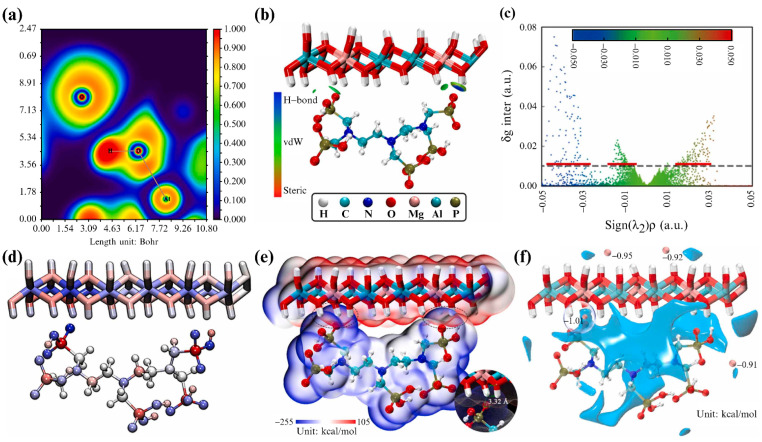
(**a**) Color-filled electron localization function diagram. (**b**) Optimized structure and independent gradient model based on a Hirshfeld partition (IGMH) map (isovalue = 0.01 a.u.). (**c**) Sign(*λ_2_*)*ρ*—colored IGMH scatter plot. (**d**) Colored atoms based on their contribution to the interaction energy. (**e**) Electrostatic potential mapping on the van der Waals surface. (**f**) Isosurface map of the van der Waals potential using C as the probe atom. Reproduced with permission from [14].

The anion intercalation driving force is the interaction force between the guest anions and the cation laminate in LDHs. It is mainly composed of electrostatic, hydrogen, and coordinate bonding [10,11], and is therefore related to the cationic composition and characteristics of the guest anions [12,13]. Recently, DFT calculations and molecular dynamics (MD) simulations have been used to approximatively estimate the interaction between the cationic layer and the guest anion. Zhu et al. [14] prepared a diethylenetriamine penta (methylene phosphonic acid) (DTPMP)-intercalated LDH composite (D−LDH), and used it as an adsorbent to remove Cr^3+^ and Cd^2+^ ions. They employed visual studies of the weak interactions to analyze and calculate the bond cooperation between the cationic laminate and the DTPMP anion (Figure 1). In the color-filled electron localization function diagram (Figure 1a), a strong H–O–Al bond was observed where the electron density region was large. In contrast, the O and H atoms of the DTPMP anion formed hydrogen bonds (Figure 1b,c). These hydrogen bonds exhibited electrostatic properties, as confirmed by the interpenetration and overlap of the negative surface potential of O in the DTPMP anion (blue area) and positive surface potential of hydrogen in the LDHs (red area) (Figure 1d,e). In Figure 1f, van der Waals forces between the anionic and cationic layers of DTPMP were observed; the probe atom was subjected to a strong dispersion gravitational driving force, with a minimum value of −1.01 kcal/mol.

**Figure 2:**
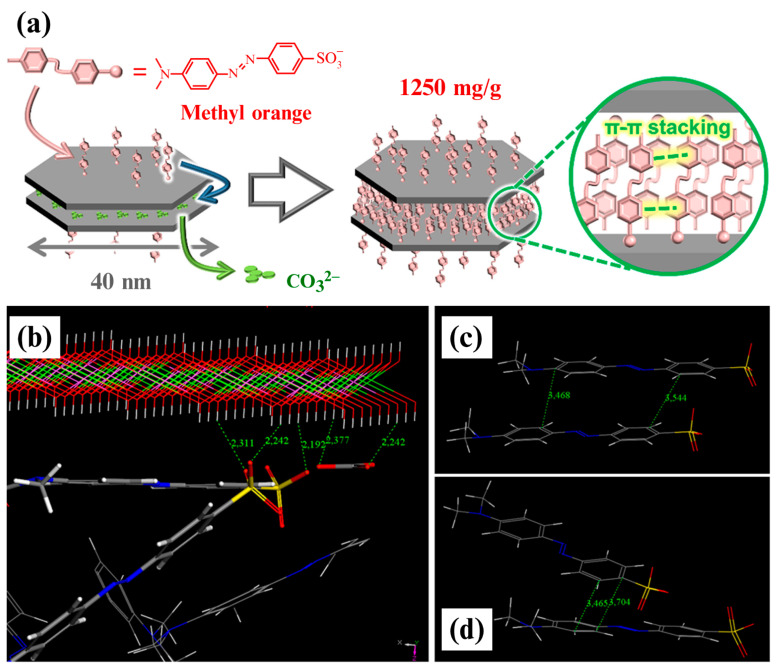
(**a**) Mechanism of the substitution of CO_3_^2−^ anions by methyl orange (MO); (**b**) snapshot of the interactions between the MO/CO_3_^2−^ and layered double hydroxide (LDH) layers and (**c**,**d**) π–π stacking of MO in an LDH simulated by the Monte Carlo method. Reproduced with permission from [17].

The influence of the anion intercalation driving force on the anion-exchange performance was first observed in the difficulty levels of various anion-exchange reactions. Conterosito et al. [15] used different stoichiometric tools (PCA, data mining, and multivariate correction) in analytical chemistry to predict the possibility of insertion into LDHs for various organic compounds. Anion-exchange reactions can only occur in LDHs when the intercalation driving force of the guest anion is greater than the interaction force between the original anion and the cation laminate [16]. Specifically, the guest anion must overcome a sufficient energy barrier to successfully exchange with the original anion. For example, it is difficult for other anions to overcome the barrier to replace CO_3_^2−^ anions and for some organic anions to replace inorganic anions [17]. Ko et al. [18] studied the unique characteristics of methyl orange (MO) intercalation in carbonated LDHs with a small transverse size and high dispersion using Monte Carlo simulation. In general, the adsorption enthalpy of CO_3_^2−^ is significantly higher than that of MO, making it difficult for MO molecules to enter the LDH interlayer. However, the smaller transverse size of the LDH provided the MO molecule with a small edge perimeter that allowed it to form π–π bonds (Figure 2a). Therefore, the MO molecule could overcome the stability of the CO_3_^2−^ anion under the combined action of electrostatic (Figure 2b) and π–π bond (Figure 2c,d) forces, thereby achieving anion replacement with the CO_3_^2−^ anions. This indicates that increasing the intercalation driving force is an effective means to promote the anion-exchange reaction.

When multiple anions coexist, anions with a higher intercalation driving force are preferentially exchanged in LDHs, which is manifested as an affinity for the anions. Zhao et al. [19] found that the anion-exchange sequence was consistent with the change in the Gibbs free energy of the anion-exchange reaction, which concurred with the experimental anion-exchange sequence. Mahmoud et al. [20] studied the adsorption performance of Zn–Al LDHs for methylene blue (MB), MO, and malachite green (MG), and found that the selective adsorption of the dyes by LDHs was in the following order: MO > MB > MG. The reason for this phenomenon is closely related to the bonding between the cationic laminates and the anions. Monte Carlo simulations showed that, in addition to electrostatic and hydrogen bonds, MO formed coordination bonds with the Zn ions in the LDH plates. In contrast, the MB molecules formed electrostatic and hydrogen bonds with the LDHs, while the MG molecules only formed electrostatic bonds. Therefore, the LDHs displayed a better affinity for MO in the environment of competitive anion coexistence. This analysis revealed that the energy barrier of the reaction is determined by the relative strength of the host–guest interaction before and after reaction, together with the balance of the initial and final anion solvation energies [21]. It is worth noting that the acid–base composition of the solution also has a significant influence on the anion intercalation driving force. For example, under basic conditions, due to the competition between the excess OH^−^ and MO anion in the solution, the intercalation driving force of the MO anion is greatly weakened, resulting in the reduction in the adsorption capacity of MO by LDHs [22]. The acidic sites of LDHs involve a large number of surface hydroxyl groups, which are more conducive to CO_2_ adsorption [23].

### 2.2. Regulation Effect of the Metal Cation Composition on the Anion Intercalation Driving Force

**Figure 3:**
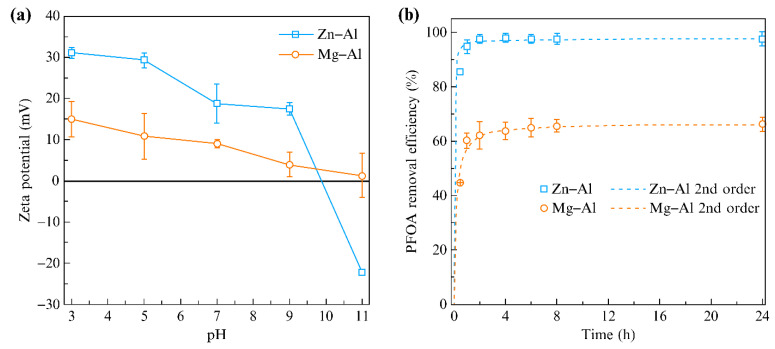

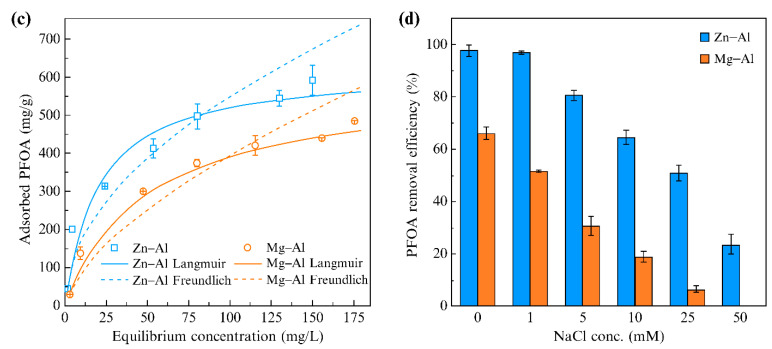
(**a**) Zeta potentials of Zn–Al and Mg–Al LDHs at pH 3–11; (**b**) adsorption kinetics of perfluorooctanoic acid (PFOA) onto the Zn–Al and Mg–Al LDHs at pH 6 with an initial PFOA concentration of 10 mg/L and adsorbent loading of 0.25 g/L. The dashed lines represent pseudo-second order model fits. (**c**) Adsorption isotherms of PFOA onto the Zn–Al and Mg–Al LDHs at pH 6 with an adsorbent loading of 0.25 g/L; (**d**) effect of the ionic strength on PFOA adsorption onto the Zn–Al and Mg–Al LDHs. Reproduced with permission from [26].

The anion intercalation driving force determines the feasibility of the anion exchange and anion selectivity of LDHs. In addition to environmental factors (e.g., temperature, concentration, and pH), the anion-exchange performance of LDHs is mainly determined by internal factors, such as the spatial arrangement of the metal cations in the laminates, charge density and distribution, and arrangement of water molecules between the laminates [24]. The contribution of each energy term to the total potential energy was analyzed, revealing that the electrostatic Coulomb force was the main driving force in anion exchange [10]. Therefore, the anion-exchange driving force can be controlled to a great extent by adjusting the charge density distribution characteristics of the cationic laminate. The charge density of laminates is regulated by changing the composition of the metal cations. For example, Li–Al LDHs prepared by the combination of monovalent and trivalent cations displayed a higher charge density in cation laminates, which led to a higher anion-exchange capacity [25]. Huo et al. [26] prepared Zn–Al LDH and Mg–Al LDH, intercalated with nitrate, and then used them as adsorbents to remove perfluorooctanoic acid (PFOA). They reported that the adsorption of PFOA on the LDHs was mainly dependent on electrostatic interaction, and the cationic composition of the LDH had a significant effect on its PFOA removal performance. Thus, owing to its higher positive zeta potential at pH 3–9 (Figure 3a), Zn–Al LDH displayed a stronger adsorption affinity, faster adsorption kinetics (Figure 3b), and a higher adsorption capacity (Figure 3c) than those of Mg–Al LDH. A series of adsorption experiments conducted at ionic strength further confirmed the importance of electrostatic interaction for PFOA removal (Figure 3d). Therefore, increasing the lamellar charge of LDHs was concluded to be a key strategy for regulating the driving force of anionic intercalation.

However, the anion-exchange performance of LDHs does not always improve with an increase in the layer charge. Many studies have proved that a higher cationic lamellar charge density may increase the interaction force between the layers and initial anions, which is not conducive to the embedding and migration of guest anions. For example, at pH 7 and T = 298 K, the maximum adsorption capacity of Ni_4_Al_1_–Cl^−^ LDH for MO was reported to be 900.84 mg/g, which is larger than that of Ni_2_Al_1_–Cl LDH, which comprises a higher lamellae charge (749.40 mg/g) [27]. A similar trend was also observed in other studies; for example, the adsorption amount of Co_4_Al_1_–Cl^−^ LDH (827.5 mg/g) was significantly greater than that of Co_2_Al_1_–Cl^−^ LDH (581.9 mg/g) [28]. This was attributed to a reduction in the percentage of Al^3+^ cations resulting in a reduction in the surface area per unit charge, which increased the electron mobility between the layers and made anion exchange easier to achieve. Matusik and Deng [29] indicated that with the decreasing charge in the cationic laminate of LDH, the content of the interlamellar binding CO_3_^2−^ also decreased, while the nonpolar sites of the cationic laminate increased, increasing the MgAl–CO_3_^2−^ LDH adsorption capacity. Thus, the electrostatic attraction competitiveness of the guest and initial anions should be comprehensively considered when adjusting the anion-exchange performance. Doubtlessly, the increase in the lamellar charge density can enlarge the retention amount of interlamellar anions; however, it also improves the electrostatic attraction of all anions indiscriminately. Whether the guest anions can successfully replace the initial anions depends more on their own competitiveness, which is manifested by the affinity of LDHs for different anions.

**Figure 4:**
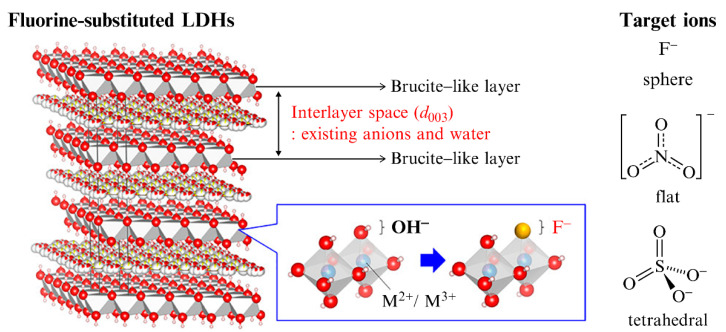
Crystal structure and schematic image of the fluorine substitution of a brucite-like layer of LDHs, visualized using the Visualization for Electronic Structural Analysis program, and molecular structures of the target ion in this study. Reproduced with permission from [33]. Copyright {2020} American Chemical Society.

Another important purpose for regulating the anion intercalation driving force is to change the affinity of LDHs for specific anions. The species of the laminate cations can influence the affinity of the anions to a certain extent. For example, the affinity of MgAl−LDHs for various anions is of the order: NO_3_^−^ < Br− < Cl^−^ < F^−^ < OH^−^ < MoO_4_^2−^ < SO_4_^2−^ < CrO4^2−^ < HAsO_3_^2−^ < HPO_3_^2−^ < CO_3_^2−^ [30]. In contrast, the affinity of Ca−Al LDHs for anions increases in the order: OH^−^ < SO_4_^2−^ < CO_3_^2−^ < Cl^−^ < NO_2_^−^ < NO_3_^−^ [31]. Costa et al. [32] reported that the exchange selectivity order of the simulated Zn_2_−Al_1_−LDHs was: NO_3_^−^ < Br^−^ < CO_3_^2−^ < Cl^−^ <F^−^ < OH^−^. However, the key factors that determine how the cation composition of LDHs affects the affinity of anions are still inconclusive. Assuming that Coulombic, hydrogen bonding, and van der Waals forces are the possible interactions in the interlayer space, the affinity of various LDHs for anions can be considered to be the result of the competition between these forces. In general, both Coulombic and hydrogen bonding forces are considered dominant. This can clearly explain why it is easier for anions with a higher charge to remain between the LDH layers, and anions with good electronegativity have higher affinity. Sudare et al. [33] prepared fluorine-substituted LDHs (F−LDHs), by partially replacing the OH structural groups of the cationic layers with fluorine atoms, and studied the influence of the hydrogen bond changes on their affinity for anions (Figure 4). They observed that the hydrogen bond interactions between the OH structural groups and water molecules, and interlayer anions, were significantly weakened by the highly electronegative fluorine atoms, which resulted in the dominance of van der Waals forces in the interlayer space of F−LDHs. Fluorine substitution also caused the structural change of the metal cation laminates and the shrinkage of the interlayer space (steric hindrance effect), which reduced the affinity of F−LDH for large size anions. The experimental results showed that the F−LDHs with Mg/Al ratios of 3.5 had a significantly high affinity for NO_3_^−^ anions, which decreased in the order: NO_3_^−^ > HPO_4_^2−^ > Br^−^ > F^−^ > SO_4_^2−^ > I^−^. This is attributed to the reduction in hydrogen bond interactions in F−LDH, and the dominance of van der Waals forces and spatial effects. This study demonstrated that the affinity of LDH for particular anions can be controlled by regulating the hydrogen bonds.

**Figure 5:**
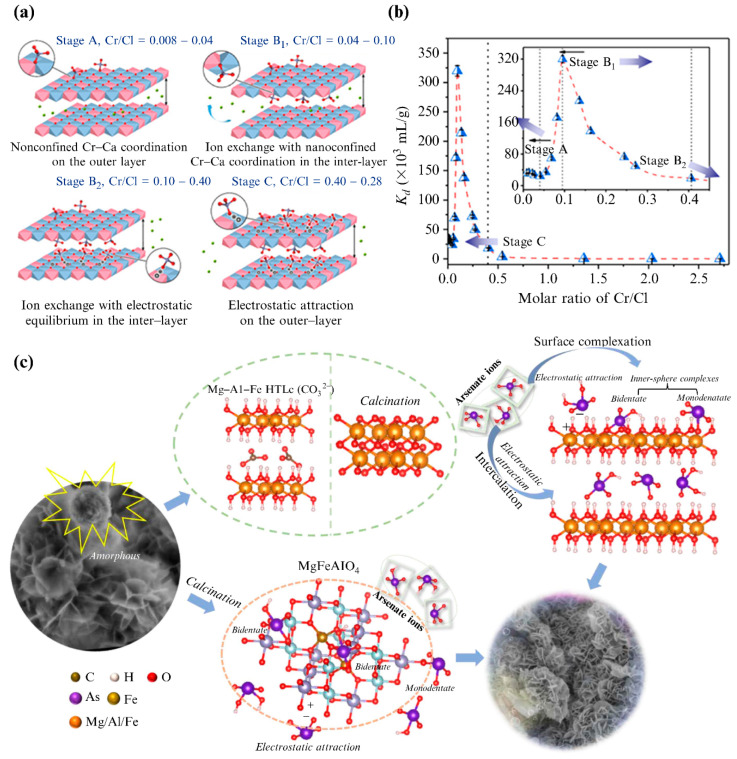
(**a**) Schematic of the binding force transformation between Cr(VI) and hydrocalumite with different Cr(VI) loadings. (**b**) Plot for the *k*_d_ values versus Cr/Cl ratios. Reproduced with permission from [40]. Copyright {2021} American Chemical Society. (**c**) Proposed As(V) removal mechanism by calcined Mg–Al–Fe LDH. Reproduced with permission from [41].

The cation coordination effect of LDH laminates can enhance the driving force of anion intercalation and improve the anion-exchange capacity [34]. When the cations on the main layer of LDHs have certain coordination properties, coordination complexes may be formed with certain anions [35,36]. If these coordination properties are adequately utilized, they can greatly enhance the anion intercalation driving force, improve the anion-exchange capacity, and even achieve selectivity for specific anions. For example, La metal cations display good phosphorus selectivity [37], and Mg/La−LDH adsorbent can selectively adsorb phosphate anions in the presence of competing Cl^−^, NO_3_^−^, and SO_4_^2−^ ions [38]. When the cationic layer has a strong coordination effect on the guest anions, the stoichiometric ratio of the released initial anions to the intercalated guest is much lower than the value calculated according to the charge balance of anion exchange [39]. Tian et al. [40] also confirmed this phenomenon, observing that the coordination effect between Ca^2+^ and CrO_4_^2−^ [Cr(VI)] ions played an important role in the strong retention to CrO_4_^2−^, which significantly increased the adsorption capacity of Ca_4_Al_2_−Cl LDH. With the increase in the Cr(VI) load, the binding force of brucite to Cr(VI) changed in four steps (Figure 5a). The affinity of the Ca_4_Al_2_−Cl LDH to Cr(VI) was increased to 3.2 × 10^5^ mL/g, owing to the Cr−Ca coordination and nano-constraint effects between the layers, which were 1–3 orders of magnitude higher than those of the ion exchange (Figure 5b). Li et al. [41] modified Mg/Al LDHs with Fe ions to prepare a flower-like nanocomposite, which still maintained a good arsenate removal efficiency in a complex anionic environment (coexisting with CO_3_^2−^, SO_4_^2−^, PO_4_^3−^, and F^−^). As shown in Figure 5c, the synthesized composite material had two forms: (1) a 3D flower-like structure assembled by nanosheets (Mg–Al–Fe LDH) and (2) a spherical nanoparticle (MgFeAlO_4_). The main adsorption mechanisms of the calcined Mg−Al−Fe LDH comprised anion intercalation driven by electrostatic forces, surface electrostatic adsorption, and surface complexation. The As(V) formed a mononuclear, monodentate ligand with Mg metal ions and binuclear, bidentate ligands with Fe/Al oxides. The composite adsorbent had high selectivity for As(V), owing to the formation of inner-sphere complexes between the metal ions of the laminate and As(V). In conclusion, the selectivity of LDHs for a particular anion can be achieved by regulating the type of laminate metal ion.

## 3. Regulation Effect of the Interlayer Domain Environment

### 3.1. Effect of the Interlayer Domain Environment on the Anion-Exchange Performance

**Figure 6:**
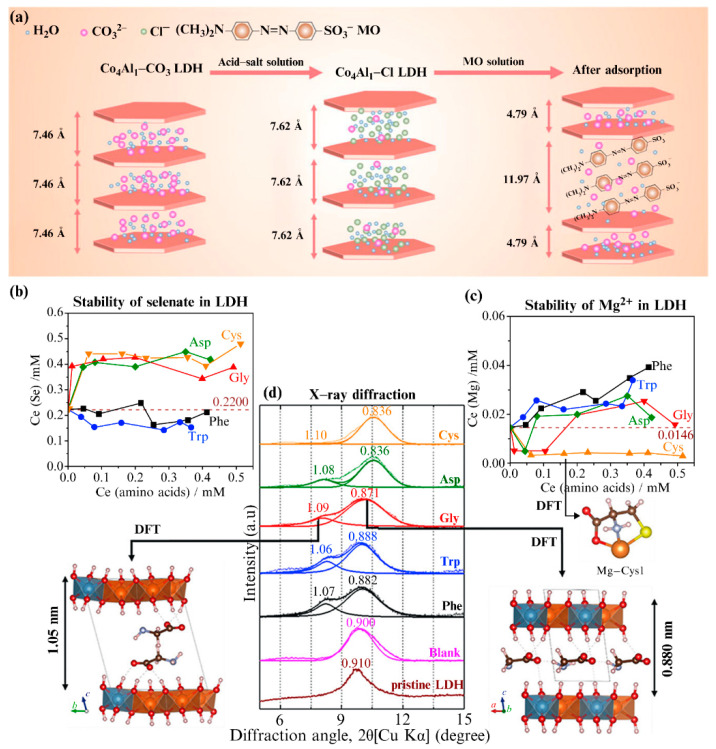
(**a**) Schematic illustration of the proposed “ion-exchange and expansion-extrusion” mechanism for MO on Co_4_Al_1_−Cl LDH. Reproduced with permission from [28]. Plots of the released concentrations of (**b**) SeO_4_^2−^ and (**c**) Mg^2+^ ions after the reaction. (**d**) X-ray diffraction (XRD) patterns of the pristine Mg_2_Al−LDH and solid residues after the sorption reaction. Reproduced with permission from [45].

The interlaminar domain of LDHs can be regarded as a structure similar to the “cavity” formed between laminates. Thus, the cationic laminate can be regarded as a “cavity wall,” wherein the thickness of the wall differs according to the different cations introduced in the laminate. The enclosed cavities between the walls have certain flexibility; i.e., the space between the layers can be flexibly adjusted according to the size, orientation, and arrangement of the anions. Owing to this variability in the LDH interlayer domain space, anion-exchange reactions can be performed smoothly, and stable structures can be obtained. Chen et al. [28] studied the adsorption characteristics of Co_4_Al_1_–Cl LDH on MO and proposed an adsorption mechanism, which they named, “ion exchange and expansion–extrusion” (Figure 6a). In their proposed mechanism, after the MO molecules enter the LDH interlayer, the thickness of the ultrathin nanosheets significantly increases, from 10–20 nm, before adsorption, to 30–50 nm, after adsorption. The interlayer spacing between the two adjacent host layers of Co_4_Al_1_−Cl−LDH is 7.62 Å. After adsorption, the MO molecules are arranged parallel or obliquely in the interlayer space, thereby expanding the interlayer spacing d_(003)_ to 11.97 Å and compressing the adjacent d_(003)_ to 4.79 Å. This expansion and extrusion of the main LDH laminate results in greater stability for the entire phase. A similar phenomenon was observed in the adsorption study of Mg_2_Al–Cl LDH [42]. Interestingly, only the anion-exchange reaction of MO displayed expansion and extrusion of the adjacent layers, while the anion-exchange reaction of Congo red (CR) and indigo carmine did not exhibit this phenomenon. The cause of this phenomenon is still unclear, and the related mechanism requires further research.

Generally, wide and flexible interlayer spaces are more beneficial to anion-exchange reactions. If the interlayer spacing is too small, the entry of large anions into the interlayer is very difficult. Thus, it is significantly difficult for large, low-charge anions to homogeneously balance the positive charge of LDH cationic laminates [43]. Costantino et al. [44] prepared ZnAl–Cl LDH, and subsequently conducted anion-exchange reactions with other halide anions (x = F, Br, and I). Isotherm analysis showed that the selectivity of this LDH toward halides decreased with an increasing ionic radius x^−^, and the selectivity was of the order: F^−^ > Cl^−^ > Br^−^ > I^−^. These results revealed that the layer spacing also has a significant influence on the affinity of the anions. Mengmeng et al. [45] studied the environmental effects of amino acids on Mg_2_Al−LDH-immobilized SeO_4_^2−^. They reported that glycine (Gly), L-aspartic acid, and L-cysteine (Cys), with smaller molecular sizes, were more easily intercalated into the interlaminar spacing, leading to the release of SeO_4_^2−^ (Figure 6b). L-tryptophan and L-phenylalanine better retained the SeO_4_^2−^ anions, owing to their larger size and aromatics. The presence of Cys stabilized Mg in the solid phase, and the corresponding density functional theory (DFT) simulation also confirmed the strong affinity between Cys and Mg^2+^ (Figure 6c). The X-ray diffraction (XRD) patterns of the solid residues after amino acid intercalation showed that the layer spacing of the Mg_2_Al LDH was partially enlarged. The layer spacing of the acromion was larger than that of the main peak, which was attributed to the double-layered superposition of the Gly molecules (Figure 6d).

The size of the interlaminar space largely determines the anion-exchange capacity, which is usually determined by the volume, quantity, orientation, and arrangement of the guest anion A^n−^. For non-spherical anions, especially the anions with long chain structures, there are several arrangements between the layers, namely parallel monolayer, parallel bilayer, inclined monolayer, and bilayer arrangements [46]. Liu et al. [30] used DFT calculations to study the interlayer structure of MgAl–A LDHs with nine different types of anions (A = Cl^−^, F^−^, I^−^, Br^−^, NO_3_^−^, OH^−^, SO_4_^2−^, CO_3_^2−^, and PO_4_^3−^) and calculated their interlayer distance. The results showed that for anions with a similar size but different valence state, e.g., NO_3_^−^/CO_3_^2−^ and SO_4_^2−^/PO_4_^3−^, the effect of anion orientation on the interlayer spacing is more significant than that of the anion charge. Li et al. [47] constructed Ni–Al LDHs containing water molecules and different anions between the layers, and then used a modified CFF91 force field from the consistent family of force fields for molecular dynamics simulation. The obtained accumulation patterns of the interlayer water molecules in the Ni–Al LDHs could be divided into three types: Type 1, comprising the interlaminar anions Cl^−^, Br^−^, OH^−^, NO_3_^−^, and CO_3_^2−^, wherein the interlaminar water molecules and anions were tightly packed and parallel to the laminates; Type 2, comprising the interlaminar anion SO_4_^2−^, wherein the distribution of the interlayer water molecules was relatively loose; and Type 3, where there was more water content between the layers and the water molecules were arranged in a double layer.

### 3.2. Regulation Effect of the Metal Cation Composition on the Interlayer Domain Environment

The adjustment of the interlayer environment by changing the interlayer spacing is an effective method to improve the anion-exchange performance, especially for large-sized anions that are difficult to replace. Evidently, as the layer spacing of the LDHs increases, more anions can be accommodated or replaced. In theory, this method to synthesize LDHs with intercalated organic molecules, to obtain a larger interlayer spacing, seems effective [48,49]; however, in practical application, especially when used as an environmental adsorbent, this type of LDH usually introduces the original organic anions into the environment and causes secondary pollution. Thus, the regulation of the LDH interlayer spacing by changing the cation composition of the laminates is considered to be a more practical and environmentally friendly method. This is based on the principle that the charge density distribution characteristics of the laminate are regulated by changing the cation composition. The repulsion between the positive charge laminates and, thus, the arrangement and number of anions between the layers can then be adjusted, and finally, the layer spacing can be changed. The laminate charge density can be controlled by changing the type, valence state, and proportion of LDH metal cations [50].

Interestingly, when increasing the charge density of the laminate to change the layer spacing, opposing results were obtained in different studies. In some studies, the increase in the charge density of the LDH laminates led to the narrowing of layer spacing, and vice versa. Li et al. [51] synthesized Ni–Al LDHs with Ni/Al ratios of 2, 3, and 4 using the hydrothermal method to obtain layer spacings of 7.62, 7.66, and 7.90 Å, respectively. They observed that the increase in the Ni/Al ratio reduced the lamellar charge, and the attraction between the lamellar layer and anions was weakened. This enabled more water molecules to enter the interlayer space to create a more open interlamellar space. Xu et al. [52] indicated that there is a strong correlation between the Cl^−^ removal rate and increase in the Mg^2+^ mole ratio in Mg–Al layered double oxide (LDO). With the increase in the divalent metal cation content, the charge density of the lamellate decreased, and the layer distance increased from 0.763 to 0.793 nm. This increase was attributed to a decrease in the interlayer anion stability and an increase in the spacing between the anions. When the n(Mg^2+^): n(Al^3+^) ratio was 6:1, the Cl^−^ removal rate was 93.06%. Everaert et al. [53] observed that the layer spacing decreased from 3.10 Å in Zn_4_–Al LDH to 2.96 Å in Zn_2_–Al LDH when preparing Zn–Al LDHs with different cation ratios. A similar trend has also been observed in Co–Al LDHs [28] and Ni–Al LDHs [27].

However, some studies have indicated that an increase in the laminate charge density leads to the generation of an electrostatic repulsion between the positive cationic laminates and expansion of the layer spacing. Huo et al. [26] observed that the layer spacing of Zn–Al LDH (0.87 nm) was larger than that of Mg–Al LDH (0.80 nm), which was attributed to the higher lamellar charge density of the former. Chen et al. [54] synthesized Mg–Al, Ca–Al, and Zn–Al LDH materials using a hydrothermal method, which displayed layer spacing values of 0.86, 0.86, and 0.89 nm, respectively. A further study on Zn–Al LDH showed that when the M^2+^/M^3+^ ratio was increased from 2 to 4, the layer spacing decreased from 0.8864 to 0.8735 nm with a decrease in the lamellar charge density. Therefore, Zn_2_−Al−LDH, with both a maximum layer spacing and a positive charge had the highest F^−^ anion-exchange capacity. Li et al. [42] observed that, under the same preparation conditions, Mg_2_−Al LDH, with a higher lamellar charge, displayed a larger lamellar spacing (7.70 Å) than that of Mg_3_−Al LDH (7.65 Å). This was attributed to the electrostatic repulsion between the laminates, which enabled a part of the Cl^−^ ions to be introduced into the interlayer with the CO_3_^2−^ anions. Because Cl^−^ was easier to replace with the guest anion, the prepared Mg_2_−Al LDH displayed a higher adsorption capacity.

The electrostatic attraction between laminates and anions and the electrostatic repulsion between cationic laminates can jointly affect the regulation of the laminate charge density on the layer spacing. The final increase or decrease in the layer spacing depends on which of the two forces is greater. Therefore, the influence of these two forces on the layer spacing should be comprehensively considered when adjusting the charge density of the laminates. According to current research data, for LDHs with CO_3_^2−^ and OH^−^ intercalation as the initial anions, the electrostatic attraction between the laminates and anions may be greater than the positive charge repulsion between the laminates, which is manifested by the reduction in the layer spacing. When the initial anion is NO_3_^−^, the lamellar charge repulsion appears to be dominant, which is manifested by an increase in the layer spacing. However, the relevant conclusions and laws still need to be verified by more experimental studies. In addition, the regulation of the interlayer spacing should also consider the anionic species introduced between the layers.

**Figure 7:**
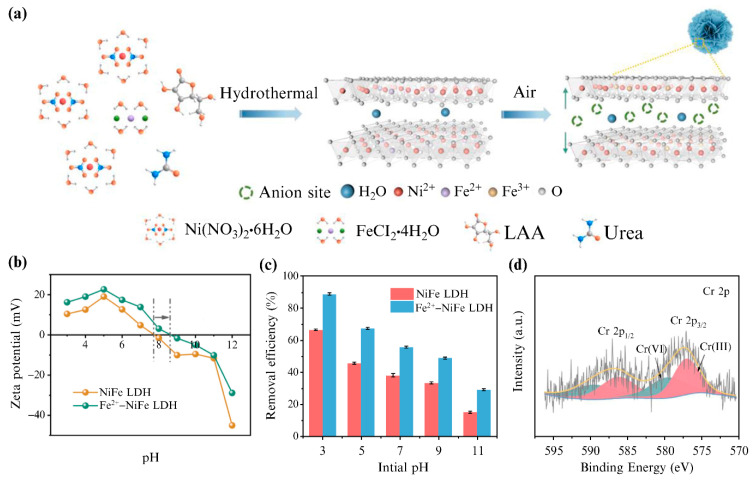
(**a**) Schematic illustration of the synthesis of Fe^2+^−NiFe LDH; (**b**) zeta potential of Ni−Fe LDH and Fe^2+^−NiFe LDH as a function of pH; (**c**) effect of the initial pH on Cr(VI) removal by Ni−Fe LDH and Fe^2+^−NiFe LDH; (**d**) Cr 2p spectrum of Fe^2+^−NiFe LDH after Cr(VI) adsorption. Reproduced with permission from [57].

The use of variable valence metal ions to adjust the electronic structure and charge distribution of LDHs can improve their catalytic activity and electrochemical performance [55,56]; however, their effect on the anion-exchange performance has been rarely studied. Huang et al. [57] improved the adsorption performance of Ni−Fe LDHs for Cr(VI) by changing their valence state to regulate the surface charge density, layer spacing, and interlayer anion charge. The Ni^2+^−Fe^2+^ LDH was synthesized by introducing L-ascorbic acid into a cationic mixed solution. The Ni^2+^−Fe^2+^ LDH then underwent topological transformation by the joint action of water and oxygen, converting into Ni^2+^−Fe^3+^ LDH, and finally forming Fe^2+^−NiFe LDH microspheres (Figure 7a). The calculations showed that the layer spacing of Fe^2+^−NiFe LDH is larger than that of Ni−Fe LDH. This was attributed to the electron transport within the cationic laminates, whereby oxidation from M^2+^ to M^3+^ led to an increase in the hydroxyl and metal–oxygen bonds, which increased the anionic sites and resulted in higher surface positive charge densities and isoelectric points (Figure 7b). The prepared Fe^2+^−NiFe LDH displayed a superior adsorption performance (Figure 7c). Apart from electrostatic attraction and anion exchange, the removal mechanism of Cr(VI) also included anion reduction coupling adsorption (Figure 7d). Cr(VI) was partially reduced to Cr(III) by the electron transfer of the hydroxyl group on the surface of the cationic laminates, and Cr(III) ions could be adsorbed on the LDHs by equal substitution complexation or coprecipitation.

**Figure 8:**
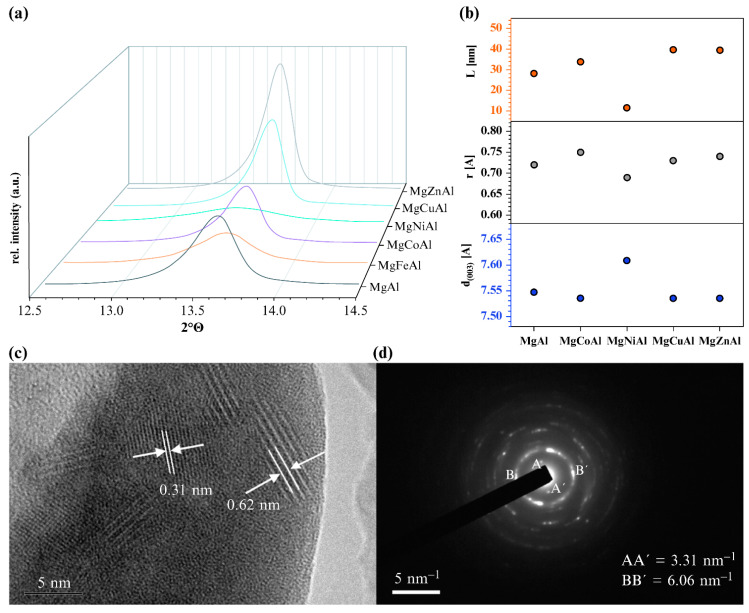
(**a**) XRD pattern of (003) reflexes for various MgMAl LDHs. (**b**) Relationship between the atomic radius, d-spacing, and grain size of the MgAl−LDHs substituted with 5% divalent transition metals. Reproduced with permission from [59]. (**c**) High resolution transmission electron microscopy (TEM) image of LaLiAl LDH. (**d**) Selected area electron diffraction pattern of LaLiAl LDH. Reproduced with permission from [60].

In addition, the geometric effect by isomorphous substitution with cations of different ion radii is also considered to be an effective means of microstructural regulation [58]. Geometric effects can lead to active site isolation, crystal structure distortion, and adsorption pattern changes, thereby increasing the activity or selectivity of the LDHs. Previous observations indicate that isomorphic replacement results in a change in the layer distance if the ionic radii of the substituted metal is quite different from that of the initial metal ion. Naseem et al. [59] synthesized transition-metal-substituted MgMAl LDHs (M = Fe, Co, Ni, Cu, and Zn), in which Mg was replaced by Ni, Co, Cu, and Zn, while Al was replaced by Fe, with the amount of substitution being 5 and 10 mol%, respectively. The surface XRD patterns of (003) peaks and the corresponding size changes of the MgMAl LDHs are shown in Figure 8a,b. Because of the different ionic radii of Al^3+^(0.535 Å) and Fe^3+^(0.645 Å), the value of d_(003)_ was increased from 7.547 to 7.572 Å by replacing Al^3+^ with Fe^3+^. Similarly, Mg^2+^(0.72 Å) was replaced by Ni^2+^(0.69 Å), increasing the layer spacing from 7.547 to 7.609 Å, even though Ni^2+^ is smaller than Mg^2+^. There was no change in the layer spacing for the substitution of Mg^2+^ (0.72 Å) with Co^2+^ (0.745 Å), Zn^2+^ (0.74 Å), and Cu^2+^ (0.73 Å), which is consistent with the fact that the four metals have similar ionic radii. This phenomenon was attributed to the change in the orientation and number of the anions (or water molecules), caused by the geometrical effect of isomorphic substitution. Cai et al. [60] indicated that La-doped Li−Al LDHs resulted in an increase in the layer spacing and a higher anion-exchange capacity. This may be because the ionic radius of La^3+^ (116 pm) is much larger than that of Al^3+^ (39 pm), and the replacement of Al^3+^ by La^3+^ would lead to the expansion of the layer spacing. Two distinct electron diffraction patterns can be observed in Figure 8c, with interlayer distances of 0.31 nm and 0.62 nm, respectively, matching the electron diffraction patterns with symmetric distances of 6.06 and 3.31 nm^−1^, respectively (Figure 8d).

## 4. Regulation Effect of the Layered Double Hydroxide (LDH) Morphology

### 4.1. Effect of the LDH Morphology on the Anion-Exchange Performance

The influence of the LDH morphology on the anion-exchange performance is significant, and this performance is improved by exposing more reaction sites and facilitating anion diffusion. Therefore, LDHs with a smaller transverse size, larger specific surface area, and more defect vacancies are beneficial for increasing the ion-exchange capacity [61,62]. Jiang et al. [63] prepared CO_3_^2−^—intercalated Ni_x_−Fe LDHs with superior adsorption performance using a coprecipitation synthesis method at a low temperature. The maximum MO adsorption capacity of this material was 416 mg^2^/g, which was significantly higher than that observed for other LDH adsorbents. This was mainly attributed to the lower crystallinity of Ni_2_Fe−LDH at low temperatures, resulting in a smaller lateral size (20–30 nm), larger specific surface area (≤427 m^2^/g), and more adsorption sites.

**Figure 9:**
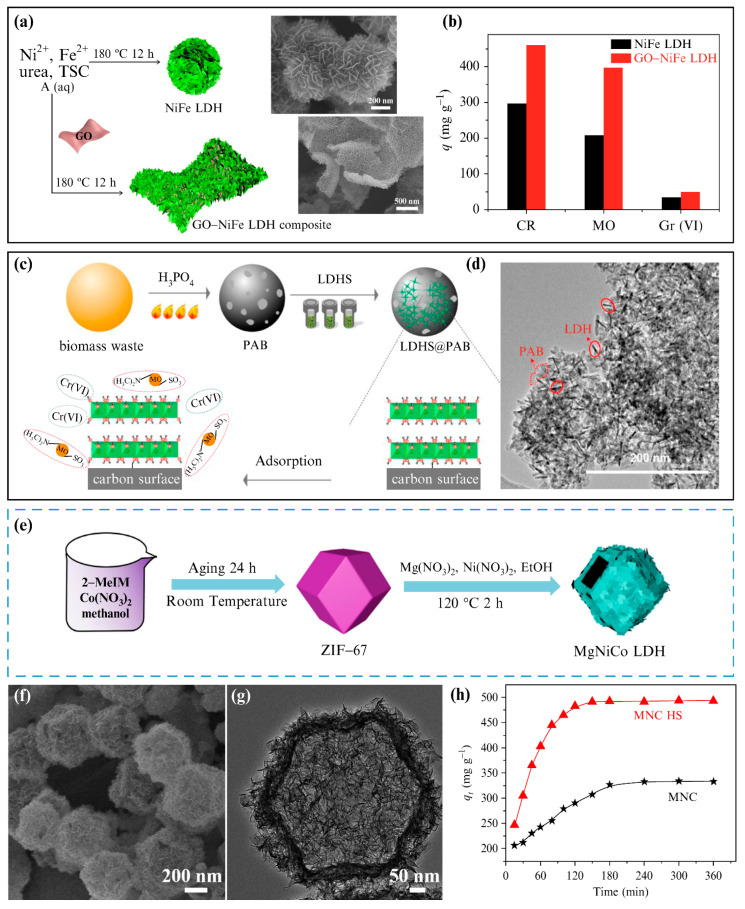
(**a**) Schematic illustration of the synthesis of graphene oxide (GO)−Ni−Fe LDH. (**b**) Absorption capacities of the Ni−Fe LDH and GO−NiFe LDH composite. Reproduced with permission from [65]. (**c**) Synthesis procedure for the LDH@biomass-derived porous carbon (PAB) composite. (**d**) TEM image of the LDH@PAB composite. Reproduced with permission from [66]. (**e**) Synthesis process of the MgNiCo LDH hollow structure (MNC HS), and its (**f**) field emission scanning electron microscopy (FESEM) and (**g**) TEM images. (**h**) Kinetic curves for Congo red (CR) adsorption on the MNC and MNC HS samples (CR concentration = 50 mg L^−1^, adsorbent dose = 0.1 g L^−1^, T = 30 °C). Reproduced with permission from [67].

In addition to the improvement of 2D nanosheets, changing the stack mode of the nanosheets and constructing the multistage structure of LDHs can further improve the anion-exchange capacity. When compared to planar crystals, LDHs with a 3D multilevel structure can: (1) provide more accessible sites for the intercalation of the given anions; (2) absorb and release a large number of alien species; (3) be intercalated, in the case of large-volume or branched anions; and (4) provide a larger surface area to generate numerous surface interactions [64]. Zheng et al. [65] also prepared 3D-layered sandwich graphene oxide (GO)−NiFe LDH composites using a one-pot hydrothermal method. The composites comprised a porous and ordered sandwich structure, which was formed by LDH nanosheets completely covering both sides of the GO (Figure 9a). The porous structure enhanced the adsorption performance. The adsorption kinetics of the sandwich GO−NiFe LDH composite were significantly faster than those of the spherical Ni−Fe LDHs, and its adsorption capacity was also significantly higher (Figure 9b). The layered, sandwich-like GO−NiFe LDH composite exhibited a good adsorption performance for MO, CR, and Cr(VI). Chen et al. [66] prepared Ni/Al@biomass-derived porous carbon (PAB) composites using a green hydrothermal method, and decorated H_3_PO_4_−activated PAB with 3D-flowered Ni/Al LDHs (Figure 9c). The Ni/Al@PAB nanocomposite displayed a unique, acicular LDH structure (Figure 9d), and good removal performances for Cr(VI) and MO in aqueous solution. Wang et al. [67] prepared a MgNiCo LDH hollow structure (MNC HS) using a one-step solvothermal method, using a metal–organic skeleton (zeolitic imidazolate framework-67 (ZIF−67)) as the sacrificial template (Figure 9e). The MNC HS retained the dodecahedral morphology and large specific surface area of ZIF−67, and also presented a hollow and porous structure (Figure 9f,g), providing abundant active sites for adsorption. Kinetic simulation showed that the adsorption capacity of MNC HS of CR was higher than the capacities of the traditionally synthesized LDHs (Figure 9h).

### 4.2. Regulation Effect of the Metal Cation Composition on the LDH Structure

In the LDH synthesis process, the composition and molar ratio of the different metal cations must be adjusted to obtain an LDH structure with good crystallinity, numerous active sites, and a large reaction area that is beneficial for ion transport. In addition to external factors, such as the synthesis methods and conditions, the internal factors that determine the structural characteristics of LDHs are the crystallization habits, which rely on their own compositions. The metal cation composition of an LDH can regulate the lamellar characteristics, nanostructure, and structural porosity of the multilevel structural units, and fundamentally determine whether the double hydroxide laminated structures can be formed [7]. This regulation effect is mainly achieved by adjusting the metal cation type, radius, and mole ratio.

#### 4.2.1. Regulation of the LDH Nanolaminates

The structure of LDH laminates can be regulated using the characteristics of the metal cation. For example, the Al^3+^ ion can stabilize the crystal lattice and improve the crystallinity of LDHs [68], while Ga acts as a structural accelerator and can cause the expansion of the lamellar lattice [69]. In Ni−Fe LDH, when Ni^2+^ ions are replaced by Fe^3+^ ions, β−Ni(OH)_2_ is converted into a layer structure that is similar to that of α−Ni(OH)_2_, forming ultrathin nanosheets with high specific surface areas [70]. Chen et al. [54] synthesized Mg−Al, Ca−Al, and Zn−Al LDH via hydrothermal synthesis, all of which displayed typical hexahedral layered morphologies. Of the three, Zn−Al LDH exhibited the best anion-exchange performance, owing to its smaller average particle size (0.23 μm), which was significantly smaller than those of Mg−Al LDH (0.99 μm) and Ca−Al LDH (0.87 μm). The addition of a third metal cation to the binary LDH system to change its optical, adsorption, and catalytic properties has also become a research hotspot in recent years. For example, replacing Mg in Mg−Al LDHs with transition metal decreased the platelet count of LDHs in the following order: Zn > Cu > Co > Mg > Ni. The platelet size decreased with the substitution of Ni, and increased with the substitution of Cu, Co, and Zn [59]. Ni et al. [71] prepared a series of Mg–Al LDOs doped with different rare-earth elements. The cation Al^3+^ (ionic radius, 0.05 nm) of the LDH laminate was replaced by Ce^3+^ (ionic radius, 0.10 nm), La^3+^ (ionic radius, 0.10 nm), and Y^3+^ (ionic radius, 0.09 nm). Owing to its smaller ionic radius, Y^3+^ could be better intercalated into the layered structure of the hydrotalcite, such that Ru/Y−LDO exhibited a smaller particle size than those of the Ce- and La-doped LDOs.

**Figure 10:**
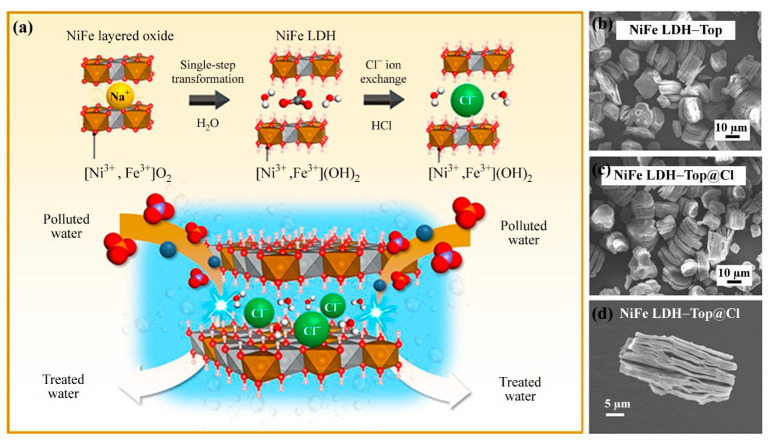
(**a**) Schematic of the fabrication and modification of NiFe LDH-Top@Cl. Scanning electron microscopy images of (**b**) NiFe LDH-Top (10 µm), (**c**) NiFe LDH-Top@Cl (10 µm), and (**d**) NiFe LDH-Top@Cl (5 µm). Reproduced with permission from [77]. Copyright {2021} American Chemical Society.

Regulating the particle size of the LDHs by changing the cation composition is another method to improve the anion-exchange performance. The principle is to reduce the size of the LDH nanosheets and weaken the interaction force between the cationic layer and initial anion, such that the guest anion can enter the interlayer more easily and form a new bond with the cationic layer [72]. In particular, the edge interlayer anions have smaller coordination numbers with the layers, so they are more exchangeable [73]. For example, stripping large LDHs into ultrathin nanosheets can produce large, exposed surfaces with active sites to induce coordination forces between water and other molecules [74,75]. Using a deep eutectic solvent, Gao et al. [76] synthesized ultrathin LDHs with small particle sizes by adjusting the ratio of cations to change the crystallinity. The results showed that the LDHs prepared in a deep eutectic solvent system, termed I−LDHs, displayed a narrow particle size distribution (10–40 nm), and the monolayer particle size was approximately 0.7 nm. As the size decreased, the interaction between CO_3_^2−^ and the metal cation laminate weakened; therefore, B^−^ could replace CO_3_^2−^, improving the boron adsorption capacity of the LDHs. Mongkol et al. [77] prepared a superior Ni−Fe LDH adsorbent using a one-step topological chemical synthesis method, as illustrated in Figure 10a. The oxidation precursor (NaNi_0.75_Fe_0.25_O_2_) underwent a configuration transformation in ultrapure water, and the valence state of the matrix layer changed from Ni^3+^ to Ni^2+^. CO_3_^2−^ ions were intercalated simultaneously to obtain a NiFe LDH-Top adsorbent (Figure 10b). The CO_3_^2−^ ions were then exchanged by Cl^−^ ions, and the final product was labeled NiFe LDH-Top@Cl (Figure 10c). Studies showed that the adsorbent had a high crystallinity, cationic state, high porosity, and unique particle shape (Figure 10d). The unique structure of this adsorbent might be attributed to the physical stress generated by intercalation and delamination during the topological transformation of the precursor, which experienced spalling in ultrapure water. The adsorbent showed a rapid and effective removal ability and high cycle stability for fluoride, phosphate, and nitrate ions.

#### 4.2.2. Regulation of the Multistage Structure of LDHs

The construction of the LDH multistage structures is the result of the combined action of many factors [78]. Among them, regulation of the electrostatic forces, coordination bonds, and van der Waals forces between the cationic laminates is key to the synthesis of LDH multistage structures. From a thermodynamic viewpoint, the formation of a 3D multistage structure is likely related to the tendency of reducing the surface energy of the system. Jose et al. [79] observed the directional attachment crystallization process of 2D-LDH nanosheets under static using liquid transmission electron microscopy. Experimental observations showed that, under static conditions, the nanosheets moved and rotated with each other in close proximity until the crystal planes were aligned and stuck together. The speed of approach is related to the alignment of the facets, while van der Waals forces are the main driving force. Related studies have also proved that LDH nanosheets prefer directional edge-to-edge [80] or face-to-face [81] assembly. Jose et al. [79] hypothesized that the charge anisotropy of LDH nanosheets is the main cause of directional attachment. The hydroxide groups on the substrate of the LDH sheet are tri-coordinated, while the hydroxide groups on the edges are di- and mono-coordinated, and thus, unstable and amphoteric, resulting in charge anisotropy. The edge hydroxyl group is more likely to attract charged groups. If the charge of the side group and Coulomb repulsion can be regulated effectively, then edge-edge nanosheet aggregation can be achieved.

**Figure 11:**
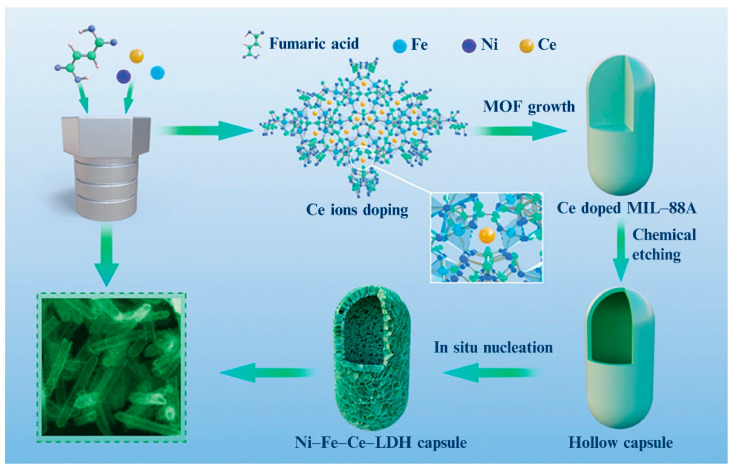
Schematic of the synthesis of hollow Ni–Fe–Ce LDH microcapsules. Reproduced with permission from [84].

The templating method is a common approach used to build 3D multilevel structures. The introduction of template guiding agents is beneficial to the formation of the multistage structure of LDHs. However, such agents are external factors that affect the mineral structure, while the crystallization habits of minerals determined by their own compositions are the internal factors that determine the structural characteristics of the minerals [82]. In fact, while synthesizing LDHs, the composition characteristics and molar ratio of the different metal cations must be adjusted to obtain an LDH structure with good crystallization performance. In some cases, the introduction of a particular metal cation has even become a key factor in the formation of 3D multilevel structures. For example, Zuo et al. [83] used a one-step hydrothermal method to prepare flower-like Ni−Co−LDH nanosheets on conductive carbon felt, with the assistance of KMnO_4_. They observed that the Ni/Co molar ratio had a significant influence on the morphology of the samples, and the coordination between the Co and Ni ions was the key to constructing the 3D, flower-like structure. During the synthesis process, the flower aggregation was rarely observed without Ni or Co addition. When the Ni/Co feed molar ratio was increased from 2:8 to 8:2, the nanosheets became increasingly thinner, with an increasing number of pores, and a clear, flower-like structure appeared. By accurately controlling the Ce^3+^ ion concentration, Xu et al. [84] synthesized a NiFeCe−LDH hollow microcapsule using a one-step solvothermal reaction. Figure 11 shows the synthesis principle diagram of hollow Ni−Fe−Ce LDH microcapsules and the important role of Ce^3+^ ions in the synthesis of the hollow microcapsules. The study found that the metal–organic frameworks formed by MIL−88A became more stable in the presence of Ce^3+^ ions, especially in weakly alkaline solutions. With the increase in the Ce^3+^ doping amount from 5 to 50%, the average length of the Ni–Fe–Ce LDH microcapsules decreased from 2 to 1 mm, and the average width increased from 200 to 300 nm. Moreover, the 3D capsule LDH could not be prepared without Ce^3+^ ions. This was because of the flexible coordination, excellent polyvalence, and high affinity of the Ce^3+^ ions for hard oxygen donors. The introduction of Ce^3+^ effectively regulated the growth process of the nanocrystals and optimized their morphology, crystal stability, and properties.

**Figure 12:**
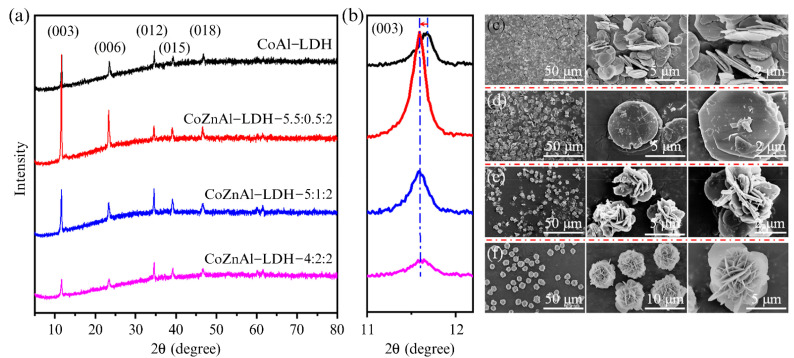
(**a**) XRD patterns; (**b**) selected XRD pattern insets of the (003) diffraction peak; and (**c**–**f**) corresponding FESEM images for CoAl−LDH, CoZnAl−LDH−5.5:0.5:2, CoZnAl−LDH−5:1:2, and CoZnAl−LDH−4:2:2, respectively. Reproduced with permission from [85].

In addition, 3D multistage structures can also be constructed depending on the coordination and charge properties of the metal cations. Yang et al. [85] built a 3D, flower-like LDH structure, by regulating the molar ratio of the cationic components in the laminates, without using surfactants or templates. The X-ray diffraction analysis showed that Zn^2+^ had a significant effect on the crystallinity of the as-produced LDHs (Figure 12a,b). It could be observed from Figure 12c–f that with the increase in the Zn^2+^ concentration, the LDH nanosheets gradually changed from 2D stacked to 3D, flower-like structures (Figure 12c–f). This change was attributed to the change in the competitive microenvironment of the Co ions in the CoAl−LDH laminates caused by the introduction of Zn^2+^ ions, which affected the growth of the crystalline structure. Similarly, without using surfactants and templates, Lei et al. [86] synthesized NiMgAl LDHs (NMA−LDHs) with a flower-like, hollow microsphere structure using a simple hydrothermal method. The maximum adsorption capacities of the as-produced NMA−LDHs for hexavalent chromium ions and CR were 103.4 and 1250 mg/g, respectively. Regretfully, these experimental phenomena have not been further studied, and the specific role and detailed causes of lamellar metal ions in the construction of 3D multistage structures currently remains unclear.

## 5. Application of the LDH Anion-Exchange Performance

### 5.1. Preparation of New Materials

It is well known that there are removable water molecules and anions in the LDH interlayer channels, showing anion exchange, hydration, dehydration, proton conduction, and other characteristics. Based on the anion-exchange method, the LDH performance can be significantly changed and improved by intercalating inorganic anions, organic acids, and organic complexes into the LDH interlayer spaces. For example, sodium dodecyl sulfate and Mo-6 clusters were intercalated into Zn−Al LDHs using an anion-exchange method. This expanded the interlayer spacings from 0.9 to 5 nm, so that the resultant material could be used as a homogeneous catalyst for the removal of organic pollutants [87]. The organic ultraviolet (UV) absorbent cinnamic acid was embedded in the interlayers of Zn−Ti LDH to create a novel UV barrier material for the host and object, which displayed a superior UV blocking ability, and thus, application potential as a sunscreen [88]. Mohammadi et al. [89] synthesized a Zn−Al LDH conversion film on an aluminum alloy surface using an in situ hydrothermal route, and subsequently intercalated diethyldithiocarbamate (DDC) molecules into the LDH interlamination to significantly improve the corrosion resistance of the matrix. Kamon et al. [90] deposited Ni−Al LDH intercalated with NO_2_^−^ anions on steel plates, using a liquid-phase deposition method, to obtain a good rust prevention effect. Chen et al. [91] prepared a TiO_2_ nanotube/Ni−Ga LDH heterostructure material using an electrochemical anodic oxidation and hydrothermal method, with potential application as a high-efficiency, photocathodic anticorrosion agent of 304 stainless steel.

More interestingly, it is an effective modification strategy to intercalate/strip block LDH into ultrathin LDHs with larger surface areas and more active sites and defects, to effectively improve the anion-exchange performance. In addition, new functional structure composites can be prepared by reassembling the stripped LDH host laminates with an anion guest. The stripping of LDH materials can be divided into two steps. The first step is to increase the interlayer distance by intercalating anions, while the second step is to peel off the layers using various techniques, such as electrostatic repulsion, laser ablation, polar solvents, and mechanical force [92,93,94]. Wei et al. [95] stripped ultrathin LDH nanosheets from the hexagonal lamellar particles of MgAl−NO_3_ LDH using an anion-exchange method. The prepared LDH nanosheets and In_2_O_3_ quantum chips were then hierarchically assembled together on a glass substrate to form multilayer films. Remarkably, the composite film displayed surface radiation characteristics that were dependent on thickness, with its lowest infrared emissivity being 0.420. The nanocomposite film displayed good infrared emission tunability and is an ideal material for energy-saving buildings and intelligent windows. Zhang et al. [96] proposed a new method for the efficient organic acid decarbonation and direct intercalation of LDHs. With the assistance of acetic acid, the interlamellar carbonates of LDHs were converted into carbon dioxide, while acetic acid was left as the interlayer anion in the gallery of LDHs. Hence, various aromatic and aliphatic organic anions, including sulfonates, carboxylates, and phosphonates, could be more easily intercalated into the gallery of the LDHs. This study provided a new pathway for the simple preparation and design of LDH hybrid materials.

In conclusion, LDH-based materials with various optical, electrical, and thermal properties can be synthesized by anion exchange and structural assembly, which are widely used in superhydrophobic membranes [97,98], flame retardant composites [99], for catalysis [100,101], and for energy storage [102,103,104].

### 5.2. Adsorbent Potential

As an adsorbent, pollutant ions can be adsorbed to LDHs through surface precipitation, electrostatic attraction, anion exchange, ligand exchange, complexation, and dissolution precipitation [105,106]. Among them, anion exchange is the main adsorption mode of LDHs and the main reason for the high adsorption capacity of these materials. Zhang et al. [107] prepared monolayers of Mg−Al LDH using an ultrasound-assisted method and showed a high adsorption efficiency for Cr(VI) in formamide. The adsorption equilibrium could be reached within 1 min and a 100% removal rate was achieved. The adsorption mechanism includes anion exchange, electrostatic attraction, and surface adsorption. Zhao et al. [108] used magnetic LDHs composed of Mg(II)−Al(III) as the adsorbent and studied the adsorption of the plant hormone, indole-3-butyric acid (IBA). The adsorption results showed that magnetic LDH-1, which was synthesized using a coprecipitation method with ammonia as the base source, presented the best adsorption capacity (maximum adsorption capacity, 522.6 mg/g). The adsorption was characterized by two stages of anion exchange. The first comprised adsorption on the outer surface of the crystal and the second, molecular exchange of IBA in the interlayer field. Yang et al. [109] used corn stalk-derived biochar as the raw material assembled with M−LDHs (M = ZnAl, MgAl, and NiFe) by the simultaneous pyrolysis of biomass waste and different metal hydroxide precipitates. The final products were named B−M−LDHs, and their adsorption properties and possible adsorption mechanism for phosphate were investigated. The study showed that the adsorption mechanism of these B−M−LDH composites for phosphorus included ion exchange, electrostatic action, and ligand exchange, among which ligand exchange was the dominant process to form the inner-sphere complex. The presence of the metal-bound hydroxyl group in the B−M−LDH−1 composite could form a complex with P. Meanwhile, in a low−pH solution, the presence of H^+^ protonated the surface, and the −OH_2_^+^ cation was beneficial to the electrostatic attraction of P.

**Figure 13:**
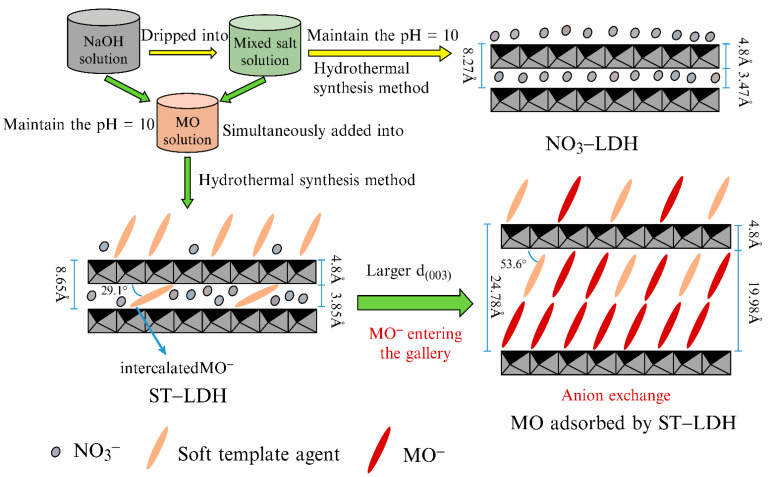
Schematic for the synthetic procedure and adsorption mechanisms of organic Zn–Cr LDH, named ST−LDH, for MO, using a one-step hydrothermal method and MO as a soft template. Reproduced with permission from [118].

Owing to the advantages of a controllable composition and structure, large adsorption capacity, no pollution, and low cost, LDH-based adsorbents have been widely applied in environmental governance. Current research focuses on the removal of heavy metal cations [110,111,112], radionuclides [113,114], and organic pollutants [115,116,117] in aqueous solutions. Lin et al. [118] used MO as a soft template and synthesized organic Zn−Cr LDH, which they named ST−LDH, using a one-step hydrothermal method. Figure 13 shows the synthesis process of the LDH material and the adsorption process of MO by ST−LDH. Notably, the maximum adsorption capacities of ST−LDH for CR, MO, and Orange II (OII) were 1.8, 1.86, and 2.32 times those of the original NO_3_−LDH, respectively. This was attributed to two main reasons. First, the structure of the ST−LDH changed from cellular to stacking after modification, which allowed for more exchangeable NO_3_^−^ ions to remain in the interlayer area. Second, the expansion of the interlamellar spacing, caused by the pre-intercalation of the benzene sulfonate column, facilitated the entry of the pollutant anions into the ST−LDH interlayer.

However, the practical applications of nanosized LDH materials are limited owing to several reasons. In particular, the agglomeration of LDH nanoparticles leads to a decrease in the adsorption capacity; moreover, small particles are not easy to separate and the pressure drop is too large when applied to the flow system. Fixing the LDH nanoparticles on the porous carriers of large particles to prepare composite adsorbents is an effective method. The LDH-based nanocomposites have been produced by the hybridization of LDHs with carbon nanotubes (CNTs) [119], reduced GO [120], carbon nanofibers [121], and other materials, and have the advantages of a large specific surface area and good adsorption capacity. Kaur et al. [122] prepared a series of g−C_3_N_4_@NiCo LDH composites by loading 10–30 wt% graphitic C_3_N_4_ (g−C_3_N_4_) onto LDHs by electrostatic self-assembly. The results showed that when the g−C_3_N_4_ loading was at 30 wt%, the adsorption capacity for MB reached 25.16 mg/g, which is 6–7-fold greater than that of the bare LDHs. Zhang et al. [123] synthesized magnetic mesoporous NiFe_2_O_4_/ZnCuCr LDH composites from saccharin wastewater using an environmentally friendly hydrothermal method and used them as adsorbents to remove Condor red from wastewater, thus achieving the purpose of “treating waste with waste”.

Based on LDH composites, the preparation of a 3D multistage structure that can expose more adsorption sites is an effective means to increasing the adsorption capacity. In general, the adsorption of positively charged nanosheets onto a negatively charged template surface by electrostatic force is a common method to obtaining multistage structures [124,125]. Lyu et al. [126] prepared a 3D LDH/carbon sphere (CS) composite using a self-assembly method. A simple and effective LDH/CS composite material was obtained, owing to the electrostatic adsorption between the LDH and the CS. After calcination, a hollow structure with a large specific surface area was formed after removing the carbon core. Simultaneously, the LDH was transformed into an LDO, which showed good negative ion adsorption capacity. The maximum adsorption capacities of amaranth and OII were 638.634 and 849.674 mg/g, respectively, under optimal adsorption conditions, which indicated that the prepared material could effectively adsorb anionic dyes, especially those with less charge.

### 5.3. Drug-Carrier Potential

Conventional drug delivery systems have limitations such as the first-pass effect, high dose requirements, and the inability to provide a continuous drug release [127]. To overcome these defects and improve the therapeutic effect of drugs, increasing attention is being focused on the research of intelligent drug delivery systems. Among the various types of nanomaterial carriers, LDH-based drug carriers have been widely studied in the treatment of many diseases [128] because of the following advantages: (1) LDHs display strong anion-exchange capacities and thus, multiple anions can be intercalated into their interlayers; (2) positively charged LDHs can carry negatively charged drugs and can be applied for targeted therapy for disease treatment; (3) LDHs have the ability to continuously release embedded drugs and improve the bioavailability of intercalated drugs; (4) owing to their high mechanical strength, they can be used to improve the mechanical strength of polymer matrices; and (5) LDHs have the advantages of high chemical stability, good biocompatibility, and pH-dependent solubility [129].

Clearly, the main reason why LDH-based drug carriers have received so much attention is that their interlayers can accommodate various types of anionic drugs. In particular, a wide variety of drugs has been intercalated into LDHs by anion exchange. These include vitamins, anticancer and antituberculosis drugs, anti-cardiovascular agents, antioxidants, antifibrinolytic agents, anticoagulants, antimycotic agents, diabetes drugs, and nonsteroidal anti-inflammatory drugs [130,131,132,133,134]. Intercalated drugs can be released by both the acidic dissolution of the LDH layers and ion exchange with surrounding anions. Wang et al. [135] showed that LDHs have a strong protective effect on the etoposide VP16, which induces the in vitro toxicity of mouse embryonic stem cells and in vivo embryonic development disorders. This provides important guidance for the clinical application of LDHs as an alternative treatment system with minimal side effects in pregnant women suffering from cancer. To expand the application range of LDH-based drug carriers, some researchers have also attempted to intercalate cationic drugs into the LDH interlayers. Kim et al. [136] intercalated the cationic anticancer drug doxorubicin (DOX) into LDHs with the anionic polymer polyacrylic acid (PAA), and investigated the anticancer activity of the DOX−PAA−LDH hybrid against human lung adenocarcinoma epithelial cells and human osteosarcoma cells. DOX and PAA were intercalated into the LDH interlayer using the electrostatic forces between the PAA carboxylate and LDH layer and between DOX and the carboxylate PAA backbone. The results showed that the hydromechanical radius of the DOX−PAA−LDH hybrid was −300 nm and its initial particle size was −160 nm, which had a good cell absorption size and could continuously provide drugs to intracellular cells during endocytosis and exocytosis.

**Figure 14:**
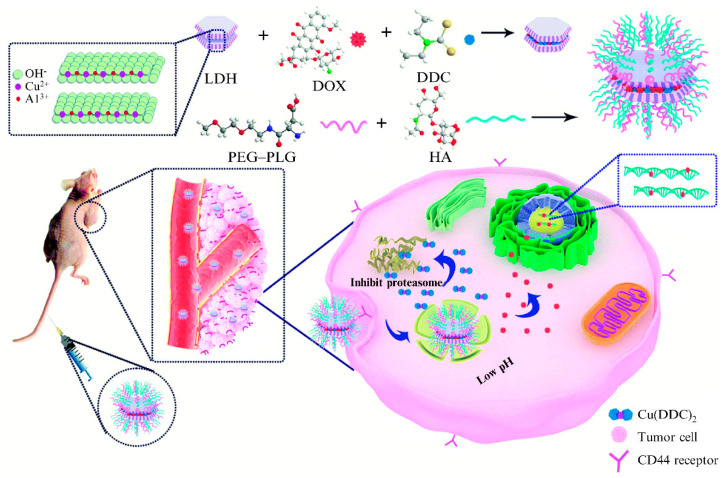
Schematic plot of the preparation, accumulation at the tumor site, and internalization into the cancer cells of hyaluronic acid/polyethylene glycol-graft-polyglutamic acid@LDHs@ diethyldithiocarbamate/doxorubicin. Reproduced with permission from [137].

In addition to acting as a carrier, the LDH itself can react with particular drug anions to become part of the drug. Xu et al. [137] synthesized CuAl−LDH nanoparticles using a coprecipitation method and intercalated DOX and sodium DDC into the interlamination of the LDHs by anion exchange. The combination of DDC and Cu^2+^ in the LDHs led to the formation of the sodium DDC−Cu complex Cu(DDC)_2_, which was not only the carrier component, but also an active ingredient in cancer treatment. The LDH was then coated with polyethylene glycol-graft-polyglutamic acid (PEG−PLG) and hyaluronic acid (HA) to enhance targeting and stability. Figure 14 shows the preparation process of the doxorubicin intercalated copper diethyldithiocarbamate functionalized LDH hybrid nanoparticles and their action mechanism at the tumor site. Experimental results showed that the HA/PEG−PLG@LDHs@ DDC/DOX nanoparticles could efficiently and effectively achieve the collaborative treatment of intractable hepatocellular carcinoma.

Improvement of the drug loading rate and loading capacity of LDH-based carriers is also a crucial problem. Owing to the large size of some drug ions and the different intercalation driving force required between different types of LDHs and various anionic drugs [138], the loading rate of drugs is significantly limited. For example, the 53.5% (*w*/*w*) loading percentage of methotrexate in the Ca−Al LDH interlayer [139] can be compared to that of Zn−Al LDH (34.5% (*w*/*w*)) [140] and Mg−Al LDH (33.2% (*w*/*w*)) [141]. Ca−Al−Ca–Al LDH, with a Ca/Al molar ratio of 3:1, can load 75.9% (*w*/*w*) of ciprofloxacin (CIP) [142]. To increase the loading capacity, Cherif et al. [143] co-intercalated CIP in its anionic form and Al^3+^ cations into the LDH interlayer to obtain a higher drug load than that attained by anion exchange alone. By strictly controlling the synthesis parameters, especially the Al^3+^/CIP ratio, two LDH−CIP intercalated structures with completely different layer spacings were obtained, i.e., 21 and 32 Å. The structure with the larger interlayer distance contained both Al(CIP)_(3)_ complexes and CIP anions, suggesting the possibility of increasing the CIP loading beyond the anion-exchange capacity of LDH carriers.

In some special disease treatment fields, such as photothermal therapy and magnetic resonance, simple LDH drug delivery systems cannot meet the needs of practical applications. Thus, it is necessary to develop an intelligent, multifunctional compound drug delivery system. Usman et al. [132] successfully synthesized a nanodrug delivery system using Zn−Al LDH as the nanocarrier, chlorogenic acid as the therapeutic agent, and gadolinium as the diagnostic contrast agent. Preliminary studies on the as-produced nanohybrid material, termed ZAGCAu, have shown its potential for the simultaneous treatment and magnetic resonance imaging diagnosis in cancer therapy. Anirudhan and Sekhar [144] combined isocyanate-functionalized LDH (LDH−NCO) with folic acid-coupled thiochitosan (TCS) to form the complex, LDH−NCO−TCS, and then added Au nanoparticles (AuNPs) to the complex surface through electrostatic and soft acid–soft base interactions to form the nanocomposite carrier, LDH−NCO−TCS/AuNP. Finally, the cancer drug DOX was loaded into the carrier through hydrogen bonding and static interactions. This composite material meets the combined therapy requirements of targeted chemotherapy and photothermal therapy in breast cancer treatment. Quantitative flow cytometry analysis exposed that the LDH−NCO−TCS/AuNP carrier generated a higher percentage of cell death at the G0/G1 phase in Michigan Cancer Foundation-7 cells.

In addition to their wide application potential as drug carriers, LDH carriers have also been studied for their potential in the cosmetic field. Cosmetics often contain active ingredients that are beneficial to the skin. However, such ingredients are sensitive to light, pH, temperature, and air oxidation, which may decrease their efficacy or cause deterioration. Because of their unique characteristics, LDHs have been proposed as an innovative carrier for the effective transfer of active ingredients to the skin. This is achieved by changing the release spectrum of the active molecules, thereby improving the stability of the activity, and increasing the solubility and bioavailability [145]. The applications of LDHs in cosmetics include: (1) controlled release ability; (2) excellent adsorption; (3) ion-exchange ability; and (4) stable potential. Of these, ion-exchange characteristics can be used to protect and stabilize unstable active components in the cosmetic formulas. Kim et al. [146] obtained well-organized, porous LDH/chitosan polymer nanocomposites by calcination reconstruction, which can be used as materials for cosmetic liposome capsules. The polymer network of the nanocomposite could effectively block liposome release, resulting in the easy absorption of large liposome particles and allowing for their controlled release. Recently, Wang et al. [147] intercalated the bacteriostatic agent and melanin synthesis inhibitor, kojic acid, into ZnTi−LDH layers through anion-exchange reactions. They reported that the material can block UV rays and effectively improve the optical and thermal stabilities of the embedded antibacterial and whitening active ingredients. Moreover, the material showed similar sustained-release characteristics to those of the kojic acid anion at pH 5 and significant L-dopa oxidation inhibition, indicating that the material has the potential in being used as a skin-whitening agent.

## 6. Summary and Outlook

Anion exchange is not only the mineralogical basic problem of LDHs but also the key in the fields of ion-exchange reaction, catalytic action, absorption, and environmental modification. The internal factors that affect the anion-exchange properties of LDHs include their Coulombic forces, hydrogen bonding, interlaminar anions, and morphology. These factors are mainly controlled by the metal cation composition of the laminate. Hence, the metal cation composition of LDHs directly affects the structural characteristics of the minerals to regulate the anion-exchange characteristics of these materials. This review summarizes the recent research work on regulating the anion-exchange performance of LDHs by adjusting the composition of the metal cations. The main points of the review are as follows:

1. The factors affecting the anion-exchange performance of LDHs were introduced. The effects of the intercalation driving force, interlayer domain environment, and LDH morphology on the anion-exchange performance (such as anion selectivity, exchange capacity, and arrangement) are proposed.
2. Corresponding to the influencing factors, the regulation of the LDH metal cation composition on the intercalation driving force, interlayer domain environment, and LDH morphology was described to achieve the regulation of the anion-exchange performance.
3. The application of the LDH anion-exchange performance in the preparation of new materials, adsorbents, and carriers was discussed. Owing to the diversity of intercalated anions, modifiability of these anions, and tunability of the interlayer space, LDHs have great potential in various fields and have attracted the attention of numerous researchers.

Although numerous studies have proved that the anion-exchange performance of LDHs can be effectively regulated by changing the cation composition of laminates, most studies still only focus on the description of experimental phenomena and results, without further research and analysis on the mechanism. The coordination between metal cations in binary (or multicomponent) systems and the resulting changes in crystal coordination structure are the key to the regulation of microcrystal structure and crystallinity. However, only the coordination relationship between metal cations in Ni–Fe system LDHs has been systematically and comprehensively concluded. Horizontal and longitudinal comparison experiments are a good method, but the research in this area is too scattered, and no regular theory has been summarized. Future work could fill this gap.

Three-dimensional LDHs are excellent, multipurpose, and multifunctional materials. Their 3D morphology affects the adsorption, diffusion, thermal stability, mechanical resistance, electrical conductivity, and surface capacity of the material. Therefore, 3D LDHs have greater development potential and prospects in new application fields than traditional LDHs. At present, the construction of 3D multistage morphology mostly relies on the guidance of template scaffolds and agents, which requires high preparation technology and posttreatment, and also increases the cost. In contrast, the construction of 3D LDHs without a template is difficult when forming a complex morphology. Therefore, simplifying the synthesis process and completely using the coordination between the template and metal cations to regulate the structure of 3D LDHs may be a future research direction.

With the expansion of the application of the LDH anion-exchange method, the quantitative analysis and calculation of anion-exchange performance is attracting increasing attention. The quantitative evaluation of the anion-exchange performance is of great significance for judging the adsorption capacity of the adsorption material, electric capacity of the electrode material, and carrying capacity of the carrier material. Nevertheless, research in this field has not received sufficient attention. In the future, the theoretical quantitative analysis of the anion-exchange performance of LDHs could be considered based on the charge density distribution of the metal cation laminate and the capacity of the interlayer space. The influence of external environmental factors (such as temperature, PH, etc.) should also be considered in the study of the theoretical quantitative analysis at the same time. Furthermore, ongoing efforts are still needed to summarize and deduce the laws and mechanisms of the quantitative evaluation of anion-exchange performance.

Overall, tremendous work has been accomplished to study the effect of metal cation composition on the anion-exchange capacity of LDHs. However, the regulation mechanism and rules of metal cation composition on anion-exchange performance and 3D structure, and the quantitative evaluation of anion-exchange performance need to be further explored. Thus, much work remains to be done.
